# A polyvalent hybrid protein elicits antibodies against the diverse allelic types of block 2 in *Plasmodium falciparum* merozoite surface protein 1

**DOI:** 10.1016/j.vaccine.2011.07.106

**Published:** 2011-10-13

**Authors:** Kevin K.A. Tetteh, David J. Conway

**Affiliations:** Department of Pathogen Molecular Biology, London School of Hygiene and Tropical Medicine, Keppel Street, London WC1E 7HT, UK

**Keywords:** Antigenic polymorphism, *Plasmodium falciparum*, Malaria vaccine, Merozoite

## Abstract

Merozoite surface protein 1 (MSP1) of *Plasmodium falciparum* has been implicated as an important target of acquired immunity, and candidate components for a vaccine include polymorphic epitopes in the N-terminal polymorphic block 2 region. We designed a polyvalent hybrid recombinant protein incorporating sequences of the three major allelic types of block 2 together with a composite repeat sequence of one of the types and N-terminal flanking T cell epitopes, and compared this with a series of recombinant proteins containing modular sub-components and similarly expressed in *Escherichia coli*. Immunogenicity of the full polyvalent hybrid protein was tested in both mice and rabbits, and comparative immunogenicity studies of the sub-component modules were performed in mice. The full hybrid protein induced high titre antibodies against each of the major block 2 allelic types expressed as separate recombinant proteins and against a wide range of allelic types naturally expressed by a panel of diverse *P. falciparum* isolates, while the sub-component modules had partial antigenic coverage as expected. This encourages further development and evaluation of the full MSP1 block 2 polyvalent hybrid protein as a candidate blood-stage component of a malaria vaccine.

## 1 Introduction

Complex antigenic polymorphisms present a significant challenge for design of a vaccine against the malaria parasite *Plasmodium falciparum*. Although partial protection offered by the current leading malaria vaccine candidate RTS,S appears not to be compromised by limited polymorphism in the pre-erythrocytic circumsporozoite protein [1], the problem of polymorphism is likely to be more important for vaccines based on blood-stage parasite proteins that are targets of naturally acquired immunity [2,3]. The extracellular merozoite that invades erythrocytes is an important target of immunity [4], and a leading vaccine candidate is the most abundant surface component, merozoite surface protein 1 (MSP1) which is expressed as a large ∼200 kDa precursor that needs to be proteolytically processed to allow merozoite maturation [5]. Antibodies to several parts of the protein can inhibit this processing [6], but most research has focused on the C-terminal region, particularly the 19 kDa C-terminal fragment MSP1-19 [7–10].

Although the N-terminal region of MSP1 has received less attention, it contains the most highly polymorphic ‘block 2’ sequences that group into three major allelic types (K1-like, MAD20-like, and R033-like) [11–16], with hybrid alleles occurring rarely [17,18]. The allele frequencies in endemic populations appear to be under balancing selection [12], and antibodies against the sequences have been associated with protection from malaria [11,12,14,19]. Allele-specific growth inhibition has been reported with an antibody-dependant cellular inhibition (ADCI) assay [13], although antibodies alone are not inhibitory except for a report of activity with one monoclonal antibody [20].

Previously, we demonstrated how an epitope mapping approach could be used to characterize the complex antigenic polymorphism seen in the K1-like block 2 repeat sequences, and employed this in the design of a single synthetic sequence termed the K1 Super Repeat (K1SR) [15]. Immunization of mice with this K1SR antigen elicited a broad antibody repertoire against *P. falciparum* isolates bearing diverse K1-like allelic types. Here we present the design and characterization of a polyvalent hybrid protein incorporating the K1SR sequence together with K1-like flanking block 2 sequences, T helper cell epitope sequences near the junction of blocks 1 and 2, and MAD20-like and R033-like block 2 allele sequences. To investigate the immunogenic contributions of each module that made up the final construct, five other sub-component constructs were designed and tested for comparative immunogenicity. Antibody responses were largely dependent on the presence of the T helper cell epitopes, and showed expected combinations of allele specificity. Antibodies to the full polyvalent hybrid protein raised in both mice and rabbits displayed a broad repertoire with serological coverage against isolates of all allelic types.

## 2 Materials and methods

### 2.1 Construction of sequences encoding MSP1 block 2 polyvalent hybrid proteins

**Fig. 1:**
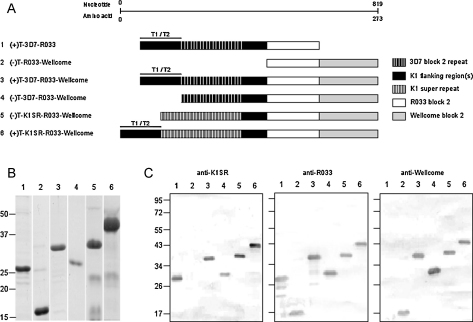
Composition of the polyvalent hybrid proteins. (A) Schematic diagram of the MSP1 block 2 constructs. Antigen 6 represents the full polyvalent immunogen with antigens 1–5 representing reagents designed and produced for comparative purposes. Each protein was expressed in *E. coli* with an N-terminal (6×)His-tag for purification. (B) Coomassie-stained 12% SDS-PAGE gel showing each of the six purified proteins (1–6 in lanes from left to right). (C) Purified polyvalent hybrid proteins demonstrate expected antigenicity on Western blots probed with murine antisera raised to GST-fusion proteins of MSP1 block 2 allelic antigens: K1 Super Repeat (K1SR), R033 and Wellcome. On each blot, lanes 1–6 contain antigens 1–6 shown in the scheme in panel A. Probing with murine antiserum raised to the 3D7 block 2 repeat antigen (not shown) gave the same pattern as for the anti-K1SR antiserum. The positions of molecular weight (kDa) markers are shown to the left.

Six recombinant antigens were constructed, five of which were designed as comparative reagents (antigens 1–5, Fig. 1A and Supplementary Fig. 1) to validate the final candidate immunogen (+)T-K1SR-R033-Wellcome (antigen 6, Fig. 1A and Supplementary Fig. 1). The DNA sequence encoding each antigen was generated using a modular construction, with each module separated by restriction enzyme sites (Supplementary Fig. 1).

For constructs incorporating the K1-like 3D7 module (antigens 1 and 3, Fig. 1A), PCR products were amplified from 3D7 parasite genomic DNA using the primer pair KTPfK1F1*Bam*H1 (5′-GGGGATCCGTAACACATGAAAGTTAT-3′) and KTPfR1*Sac*1M1 (5′-GGGAGCTCGCTTGCATCAGCTGGAGG-3′). This module also included the sequence for a conserved T-cell epitope within MSP1 block 1 (T1, amino acid position 20–39: VTHESYQELVKKLEALEDAV) and a polymorphic T-cell epitope (T2, amino acid position 44–63: GLFHKEKMILNEEEITTKGA) [21], spanning the junction of blocks 1 and 2. The R033-type block 2 module was amplified from R033 parasite genomic DNA using the primer pairs KTPfR033F1*Sac*1M2 (5′-GGGAGCTCAAGGATGGAGCAAATACT-3′) and KTPfR033R1*Kpn*1M2 (5′-GGGGTACCACTTGAATCATCTGAAGG-3′). The Wellcome (MAD20-type) module was amplified from Wellcome parasite genomic DNA using the primer pair KTPfWellF1*Kpn*1M3 (5′ GGGGTACCAATGAAGGAACAAGTGGA-3′) and KTPfWellR2*Sma*1M3 (5′-GGCCCGGGTTAACTTGAATTATCTGAAGG-3′). All PCR amplifications were performed using Accuzyme High Fidelity DNA Polymerase (Bioline Ltd, London, UK) on *P. falciparum* genomic DNA isolated from cultured parasites using the QIAamp DNA blood minikit following manufacturer's instructions (Qiagen, WestSussex, UK).

The remaining three modules were commercially synthesised (GeneArt, Germany) as codon optimized sequences for *E. coli* expression and cloned into the pG4 shuttle vector. These were: (i) a 3D7 allelic block 2 module that lacked the N-terminal T cell epitopes (in antigen 4, Fig. 1A and Supplementary Fig. 1); (ii) the K1SR module [15] also lacking the N-terminal T1/T2 T-cell epitopes (in antigen 5, Fig. 1A and Supplementary Fig. 1); (iii) the K1SR module [15] integrating the N-terminal T-cell epitopes (in antigen 6, Fig. 1A and Supplementary Fig. 1).

### 2.2 Plasmid cloning and recombinant protein expression

All synthetic DNA products were first cloned into the pGEM-T Easy cloning vector plasmid (Promega, UK). Sequence verified DNA was excised from the relevant clones using module specific restriction sites and ligated into pGEM-T Easy vector to derive the completed recombinant constructs. The commercially synthesised modules were excised using module specific restriction sites directly from the pG4 shuttle vector and cloned onto the pGEM-T backbone to derive the relevant polyvalent constructs. All constructs were sequenced at each stage to ensure fidelity of the cloned products with ABI BIGDYE terminator v3.1 chemistry using an ABI 3730xl electrophoresis system (Applied Biosystems, UK).

Each completed coding region was excised using *BamHI*/*KpnI* restriction sites for the full polyvalent hybrid protein sequence (antigen 6), and *BamHI*/*SmaI* for the remaining 5 modular polyvalent sequences (Fig. 1A), before cloning into complementary digested sites in the pQE30 His-tag expression vector (Qiagen) for antigens 1–3 or the pET15b His-tag expression vector (Novagen) for antigens 4–6 (Fig. 1A). Each cloned recombinant plasmid was transformed into M15 [pREP4] host *E. coli* strain (Qiagen) for the pQE30 cloned products or BL21 (DE3) (Stratagene) for the pET15b cloned products. All constructs were sequenced to ensure complete fidelity.

For protein expression, isopropyl-ß-d-thiogalactopyranoside (IPTG) was added to each culture to a final concentration of 1 mM following bacterial culture growth to OD_600_ of 0.6–1.0. Bacterial cells were pelleted, resuspended in BugBuster protein extraction reagent (Novagen, Merck Chemicals International) and incubated at room temperature for 20 min on a rolling platform. Cellular debris was pelleted by centrifugation, and the histidine-tagged protein purified from each supernatant following Nickel His-tag affinity chromatography using Ni-NTA agarose (Qiagen). The stability of 50 μg batches of lyophilized full polyvalent hybrid protein was tested by incubation at −20, 4, 37 and 56 °C for a period of three weeks.

### 2.3 SDS PAGE and Western blot analysis

The purified polyvalent hybrid proteins were separated under reducing conditions by 12% Tris–glycine–SDS PAGE and electrophoretically transferred to nitrocellulose membrane (Whatman, UK). Western blots were probed using murine sera raised to recombinant proteins based on the individual MSP1 block 2 types [11,15]. Bound antibody was detected with horseradish peroxidase-conjugated rabbit anti-mouse secondary antibody (DAKO), and bands visualized using 5 ml per blot of stabilized TMB (3,3′,5,5′-tetramethylbenzidine) substrate (Promega, UK).

### 2.4 Murine polyclonal sera

Groups of five CD-1 outbred mice were immunized (Northwick Park Institute for Medical Research, UK) with each antigen formulated in the ImjectAlum adjuvant (Perbio Science, Cheshire, UK). Each polyvalent hybrid protein was diluted with phosphate-buffered saline (PBS) to a concentration of 1 mg ml^−1^, and 3 volumes of Imject Alum added and allowed to mix for 30 min at room temperature. Each antigen–adjuvant mixture was administered intra-peritoneally, each mouse receiving 50 μg protein per dose in a final volume of 200 μl. Three doses were administered at monthly intervals, and blood was collected before immunization and 2 weeks after each dose (on days 14, 42, and 70).

### 2.5 Rabbit immunizations

The purified polyvalent hybrid antigen (+)T-K1SR-R033-Wellcome (antigen 6, Fig. 1A) was used to immunize New Zealand white rabbits (Pettingill Technology Limited, UK). Five rabbits received 200 μg of purified protein intramuscularly at days 0, 14, 28, 42, 56 and 70 following a 77 day protocol with one rabbit receiving adjuvant with PBS only as a control (Freund's complete adjuvant was used on day 0 immunization, Freund's incomplete adjuvant for boosting immunizations). Test bleeds were taken on days 35, 49 and 63, final bleeds were collected on day 77.

### 2.6 Indirect immunofluoresence assay (IFA) of *P. falciparum* isolates

Ten *P. falciparum* isolates were cultured, including 6 with K1-like MSP1 block 2 sequences (3D7, T9/96, T9/102, D6, K1, Palo Alto), 3 with MAD20-like block 2 sequences (Wellcome, MAD20, Dd2), and R033 representing the R033-like block 2 type that has minimal subtypic polymorphism. Each was identified and discriminated by sequencing of MSP1 block 2. Parasite cultures were grown under standard conditions to a parasitemia of 4–10% (predominantly schizont stage although asynchronous) and cells washed twice after centrifugation before resuspension in PBS/1% BSA, for preparation of IFA slides. Specific antibody reactivities to each of the parasite isolates were assessed following previously described methods [22]. Parasites were air-dried onto multiwell IFA slides (Hendley, Essex, UK), fixed with 4% formaldehyde and tested with serial doubling dilutions of murine sera (1/50 to 1/409,600) in PBS with 1% bovine serum albumin (15 μl/well) and incubated for 30 min. Biotinylated anti-mouse or anti-rabbit IgG (Vector Laboratories Inc., California, USA) was used as the secondary antibody at a 1/500 dilution and incubated for 30 min, followed by a final 30 min incubation with fluorescein-labeled streptavidin (Vector Laboratories, Inc., California, USA) at 1/500. Slides were mounted in Vectashield mounting medium with 4′,6′-diamidino-2-phenylindole (DAPI) (Vector Laboratories, Inc., California, USA) and examined with a Nikon eclipse E600 fluorescence microscope with 100× oil immersion objective and 10× eyepiece. Endpoint titre for each serum was defined as the highest dilution that resulted in bright and clear schizont-specific fluorescence.

### 2.7 Enzyme-linked immune-sorbent assay (ELISA)

Sera from immunized mice and rabbits were assayed for reactivity to recombinant GST-fusion proteins previously described [23] representing each of the three MSP1 block 2 allelic types, 3D7 (K1-like), Wellcome (MAD20-like), and R033 by ELISA following methods previously outlined in detail [15,24]. Briefly, Immulon 4HBX flat bottomed plates (Dynex Technologies inc.) were coated with 50 ng/well of each recombinant protein in 100 μl of coating buffer (15 mM Na2CO3, 35 mM NaHCO3; pH 9.3). Plates were incubated overnight at 4 °C, washed with PBS-T (PBS with 0.05% Tween), blocked (1% skimmed milk in PBS-T) for 5 h and washed again. Sera were diluted (1/1000 for murine sera and 1/2000 for rabbit sera) in blocking buffer, and 100 μl volumes were aliquoted in duplicate into antigen coated wells and incubated overnight at 4 °C. Plates were washed and wells incubated with either rabbit anti-mouse (P0260, Dako UK) (1/5000 dilution) or swine anti-rabbit HRP-conjugated IgG (P0399, Dako UK) (1/4000 dilution) for 3 h at room temperature. Plates were washed and developed with O-phenylenediamine dihydochloride (OPD) using SigmaFast OPD tablets (Sigma, UK). Detection of mouse IgG subclasses followed the same protocol, except biotin-conjugated polyclonal goat anti-mouse antibodies to murine IgG subclasses were used as the secondary antibody (Cambridge Bioscience, UK), followed by detection with HRP-conjugated streptavidin (Sigma, UK).

## 3 Results

### 3.1 Confirmation of antigenic composition of the polyvalent hybrid proteins

All six new recombinant proteins (Fig. 1A) were expressed as soluble products that appeared as single bands on SDS-PAGE gels (Fig. 1B), and Western blots were probed with specific polyclonal sera previously raised to GST-expressed proteins expressing the K1 Super Repeat [15] and individual block 2 alleles [23] (Fig. 1C). The individual sera reacted with predicted specificity against the different hybrid antigens, verifying the modular antigenic composition of each hybrid construct. The yield for the full polyvalent hybrid protein (antigen 6) averaged ∼13 mg/l of culture, and the lyophilized product was stable at temperatures ranging from −20 to 56 °C for at least 3 weeks.

### 3.2 Immunogenicity of the polyvalent hybrid proteins

**Fig. 2:**
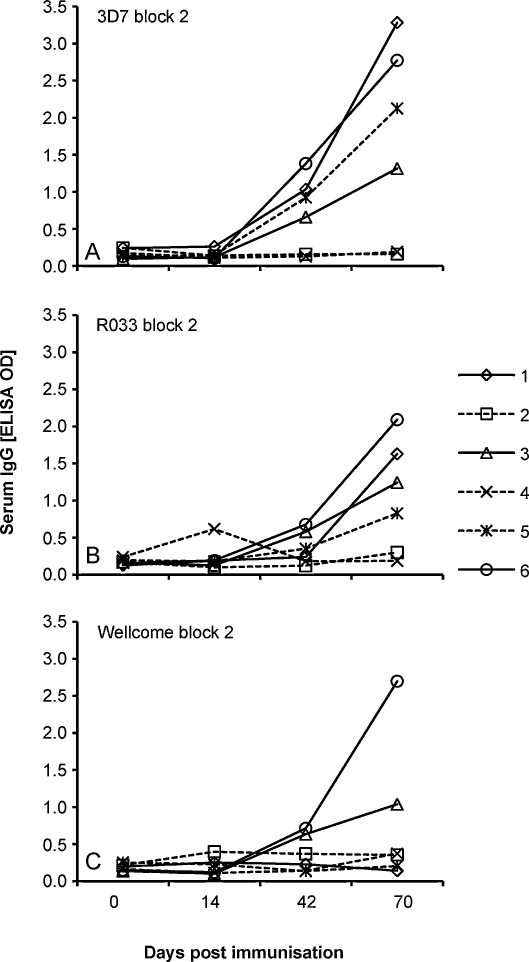
Development of parasite specific IgG responses after immunization of groups of five CD-1 outbred mice with each of the 6 polyvalent hybrid proteins (antigens 1–6 as shown in the scheme in Fig. 1A). Mean OD values for each group of mice are shown for sera from each time point assayed by ELISA at 1/1000 dilution against GST-fusion proteins representing each of the major allelic types of MSP1 block 2: (A) 3D7 (K1-like), (B) R033, (C) Wellcome (MAD20-like).

CD-1 outbred mice were immunized with each of the 6 hybrid constructs (antigens 1–6, Fig. 1A) in Alum. ELISAs were performed to determine IgG antibody reactivities against different GST-fusion proteins (MSP1 block 2 of 3D7, R033 and Wellcome alleles) [11] in sera collected from the mice at days 0, 14, 42 and 70 post immunization. The full polyvalent hybrid protein (antigen 6) elicited strong antibody responses against each of the three types of block 2 proteins, with overall higher titres and broader specificity than those elicited by the comparative polyvalent proteins (Fig. 2). Antigens that did not contain the T helper cell epitopes elicited minimal responses to the block 2 antigens except for antigen 5 that elicited some response to the 3D7 and R033 antigens. As with the murine responses, sera from each of five rabbits immunized with the full polyvalent hybrid protein showed antibody reactivity against each of the 3D7, R033 and Wellcome block 2 recombinant antigens when tested by ELISA (data not shown).

**Fig. 3:**
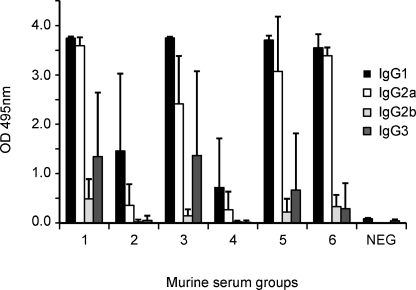
Murine immunoglobulin subclass ELISA showing the mean (+S.D.) reactivities as OD values of sera from groups of five mice immunized with the polyvalent hybrid proteins (antigens 1–6 as shown in the scheme in Fig. 1A), assayed at 1/1000 dilution against the full polyvalent hybrid protein (antigen 6). Sera were from the final bleed (day 70) and mice that were immunized with adjuvant only are compared as ‘NEG’ controls.

To test if there was a skew towards particular murine IgG subclass responses, each serum was tested against the full polyvalent hybrid protein (antigen 6) by ELISA. The responses elicited by each of the six immunizing antigens contained a predominance of IgG1 and IgG2a, rather than IgG2b or IgG3 (Fig. 3).

### 3.3 Immunofluorescence assay of different parasite lines

**Fig. 4:**
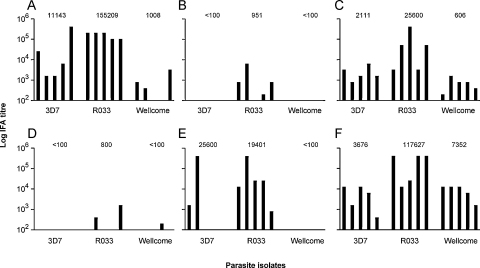
IFA endpoint titres of antisera from mice immunized with each of the six polyvalent hybrid proteins against schizonts of 3 *P. falciparum* lines, 3D7, R033, and Wellcome. The six panels A–F represent the responses of mice immunized with each of the six polyvalent antigens 1–6 respectively: A, (+)T-3D7-R033; B, (−)T-R033-Wellcome; C, (+)T-3D7-R033-Wellcome; D, (−)T-3D7-R033-Wellcome; E, (−)T-K1SR-R033-Wellcome; F, (+)T-K1SR-R033-Wellcome. The geometric mean titres of each group are indicated above the groups of five individual mouse sera.

Murine antibodies induced by each polyvalent hybrid protein were tested against cultured *P. falciparum* lines each containing a distinct block 2 allelic type, 3D7 (K1-like allele), Wellcome (MAD20-like), and R033 (Fig. 4A–F, respectively showing titres in animals immunized with antigens 1–6). Murine antibody responses to the full polyvalent hybrid protein (antigen 6) showed high titre reactivity by IFA to the three different parasite isolates (Fig. 4F). Mice immunized with the remaining five comparative polyvalent antigens produced antibodies reactive with at least one block 2 allele, but failed to achieve a similar high titre response against all three isolates (Fig. 4A–E). Antigens 2, 4 and 5, each missing the N-terminal T-cell epitopes, elicited poor antibody responses, although these were higher against R033 than against the other isolates (Fig. 4B, D and E).

**Fig. 5:**
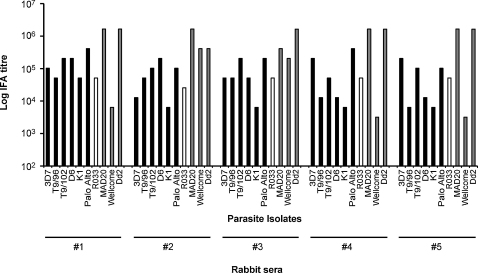
Log IFA endpoint titres of antisera from rabbits immunized with the full polyvalent protein (antigen 6, (+)T-K1SR-R033-Wellcome), against schizonts of a panel of 10 *P. falciparum* lines. The IFA titres for individual final bleed rabbit antisera (animals 1–5) are shown against the panel of parasites with MSP1 block 2 types that are K1-like (black bars), R033 (white bars) and MAD20-like (grey bars).

Sera from rabbits immunized with antigen 6 were tested against an expanded panel of 10 *P. falciparum* isolates with more diverse alleles (containing more representatives of the K1-like and MAD20-like types) (Fig. 5). Each of the five sera showed strong IFA reactivity against all isolates, with titres ranging from 1/3200 to 1/1,638,400 (Fig. 5). The titres were expected to be higher than those elicited in the mice, due to the use of a multiple immunization schedule with Freund's adjuvant in the rabbits.

## 4 Discussion

Antigenic diversity and poor immunogenicity of candidate malaria antigens present significant hurdles for the development of malaria vaccines. Inadequate design could potentially lead to survival and selection of parasites with heterologous alleles not covered by a vaccine construct [25,26]. Hybrid recombinant protein subunit vaccines are one promising approach to circumventing these hurdles. Hybrid proteins as malaria vaccines have been advocated when combining two or more unrelated proteins [27–29]. However, multi-allelic formulations for individual antigens have until now involved mixture of different allelic protein components, although viral vectors expressing a fusion of different allelic types show promise, as illustrated by work on the apical merozoite antigen 1 [30–33].

We describe the first polyvalent hybrid protein immunogen to be shown capable of eliciting a broad, high titre antibody repertoire against all major alleles of a highly polymorphic malaria antigen, in this case the block 2 region of MSP1 in *P. falciparum*. Sera of all immunized mice and rabbits recognized purified allelic recombinant antigens and schizonts of diverse parasite isolates by IFA. Importantly, incorporation of a complex composite repeat sequence to cover subtypic variation within the K1-like type [15] did not reduce the titres of antibodies to the other components.

To enhance the development of high titre antibodies to the polyvalent hybrid we included two previously described T-cell epitopes located within the N-terminal region of MSP1 [21,34]. By comparing antibody titres elicited by the modular sub-component antigens with the full polyvalent construct, it was evident that inclusion of the T-cell epitopes significantly enhanced the immunogenicity. Mice immunized with each of the constructs elicited a mixed subclass IgG1 and IgG2a response, suggesting the involvement of T helper cells of both Th1 and Th2 subsets. Such responses are generally adjuvant dependant [35,36], and the murine responses in this study were obtained with Alum that is suitable for human use.

Further work on the candidacy of this immunogen is warranted, which could include prime-boost experiments testing immunogenicity of the polyvalent sequence engineered in viral vectors as well as in the protein form described here [33,37]. It would be ideal to also have a validated assay that could be applied to test animal antibodies for parasite growth inhibition [38,39], but inhibitory effects of antibodies to MSP1 block 2 appear to require co-operation with monocytes [13] in an assay that is challenging to standardise and replicate in different laboratories [39]. In contrast, direct inhibitory effects of anti-MSP1 block 2 antibodies alone have generally not been detected [13] except in one report of a monoclonal antibody used at high concentration [20], and our attempts using well defined allele-specific rabbit antibodies unexpectedly showed non-allele-specific inhibition when tested against a panel of parasite isolates (data not shown). We anticipate that new approaches may allow further development of sensitive and specific tests for direct inhibitory effects of antibodies in the future [40]. Currently, as a pre-clinical test of the efficacy of this vaccine candidate, it would be most valuable to perform small scale immunization and challenge experiments in a new world monkey model as has been used to evaluate other individual antigens [32,41–44]. In addition to the development of a candidate for further testing, this study illustrates an approach that could be employed in the design of polyvalent immunogens based on other antigens with complex allelic polymorphisms.
