# Supplementary material for: A polyvalent hybrid protein elicits antibodies against the diverse allelic types of block 2 in Plasmodium falciparum merozoite surface protein 1
Source: Vaccine. 2011 Oct 13;29(44):7811–7. doi: 10.1016/j.vaccine.2011.07.106 (PMC3195258; doi:10.1016/j.vaccine.2011.07.106)
Supplement: Supplementary file 1 [file mmc1.pdf]

Supplementary Figure 1.

Antigen 1 [(+)T-3D7-R033] [pQE30]

[HHHHHH]**GS**VTHESYQELVKKLEALEDAVLTGYSLFQKEKMVLNEEEITTKGASAQSGASAQSGASAQSGASAQSGASAQSGASAQSGTSGPSGPSGTSPSSRSNTLPRSNTSSGASAPADAS**EL**KDGANTQVVAKPADAVSTQSAKNPPGATVPSGTASTKGAIRSPGAANPSDDSS**GT[**PGRPAAKL]**.**

Antigen 2 [(-)T-R033-Wellcome] [pQE30]

[HHHHHHGSAC]**EL**KDGANTQVVAKPADAVSTQSAKNPPGATVPSGTASTKGAIRSPGAANPSDDSS**GT**NEGTSGTAVTTSTPGSKGSVASGGSGGSVASGGSVASGGSVASGGSVASGGSGNSRRTNPSDNSS**.**

Antigen 3 [(+)T-3D7-R033-Wellcome] [pQE30]

[HHHHHH]**GS**VTHESYQELVKKLEALEDAVLTGYSLFQKEKMVLNEEEITTKGASAQSGASAQSGASAQSGASAQSGASAQSGASAQSGTSGPSGPSGTSPSSRSNTLPRSNTSSGASAPADAS**EL**KDGANTQVVAKPADAVSTQSAKNPPGATVPSGTASTKGAIRSPGAANPSDDSS**GT**NEGTSGTAVTTSTPGSKGSVASGGSGGSVASGGSVASGGSVASGGSVASGGSGNSRRTNPSDNSS**PG[**STCSQA]**.**

Antigen 4 [(-)T-3D7-R033-Wellcome] [pET15b]

[HHHHHHSSQLVPRQS]**HM**SAQSGASAQSGASAQSGASAQSGASAQSGASAQSGTSGPSGPSGTSPSSRSNTLPRSNTSSGASPPADAS**EL**KDGANTQVVAKPADAVSTQSAKNPPGATVPSGTASTKGAIRSPGAANPSDDSS**GT**NEGTSGTAVTTSTPGSKGSVASGGSGGSVASGGSVASGGSVASGGSVASGGSGNSRRTNPSDNSS**.**

Antigen 5 [(-)T-K1SR-R033-Wellcome] [pET15b]

[HHHHHHSSQLVPRQS]**HM**SAQSGASAQSGASAQSGTSAQSGTSGTSAQSGTSGTSGTSGASAQSGTSGPSGTSGTSGPSGPSGPSGPSGTSGPSGTSPSSRSNTLPRSNTSSGASPPADAS**EL**KDGANTQVVAKPADAVSTQSAKNPPGATVPSGTASTKGAIRSPGAANPSDDSS**GT**NEGTSGTAVTTSTPGSKGSVASGGSGGSVASGGSVASGGSVASGGSVASGGSGNSRRTNPSDNSS**.**

Antigen 6 [(+)T-K1SR-R033-Wellcome] [pET15b]

[HHHHHHSSQLVPRQS]**HM**VTHESYQELVKKLEALEDAVLTGYSLFQKEKMVLNEEEITTKGASAQSGASAQSGASAQSGTSAQSGTSGTSAQSGTSGTSGTSGASAQSGTSGPSGTSGTSGPSGPSGPSGPSGTSGPSGTSPSSRSNTLPRSNTSSGASPPADAS**EL**KDGANTQVVAKPADAVSTQSAKNPPGATVPSGTASTKGAIRSPGAANPSDDSS**GT**NEGTSGTAVTTSTPGSKGSVASGGSGGSVASGGSVASGGSVASGGSVASGGSGNSRRTNPSDNSS**.**

Supplementary Figure 1. Amino acid sequences showing the final candidate immunogen (Antigen 6) with the five comparative reagents (antigens 1-5). Green shading highlights the K1 amino acid sequence (antigens 1, 3 & 4: 3D7; antigens 5 & 6 K1SR). The T1 (conserved) and T2 (semi-conserved) T cell epitopes are underlined. Red and blue shading highlight R033 and Wellcome block 2 sequences respectively. Restriction sites within the hybrid coding sequences are shown in bold (BamHI = GS; SacI = EL; KpnI = GT; SmaI = PG and NdeI = HM). Vector specific sequences are shown within parentheses [ ].
